# Cell Viability of the Administered Product Predicts Response to Stromal Vascular Fraction in Patients with Erectile Dysfunction Unresponsive to Phosphodiesterase Type 5 Inhibitors

**DOI:** 10.3390/medicina62101966

**Published:** 2026-10-11

**Authors:** Ahmet Semih Güleser, Atakan Eren, Fatih Altunrende, Ufuk Çağlar, Faruk Özgör

**Affiliations:** 1 Department of Urology, Private Urology Practice, 34158 Istanbul, Türkiye; 2 Private Urology Practice, Oskar Ville Ofisleri, Hürriyet Mahallesi, Necati Sezen Sokak, Kat 1, Süleymanpaşa, 59100 Tekirdağ, Türkiye; 3 Department of Urology, Istanbul Yeni Yüzyıl University, 34010 Istanbul, Türkiye; 4 Department of Urology, Istanbul Haseki Training and Research Hospital, University of Health Sciences, 34668 Istanbul, Türkiye

**Keywords:** erectile dysfunction, stromal vascular fraction, cell viability, regenerative therapy, phosphodiesterase type 5 inhibitors, adipose-derived cells

## Abstract

*Background and Objectives*: Stromal vascular fraction (SVF) is increasingly used for erectile dysfunction (ED), but published human data are very limited and no study has related the cellular composition of the administered product to clinical outcomes. We evaluated the outcomes of SVF added to phosphodiesterase type 5 inhibitors (PDE-5I) in patients unresponsive to PDE-5I alone, and identified factors associated with treatment response. *Materials and Methods*: Records of 93 consecutive patients with vasculogenic ED who underwent autologous SVF injection in addition to PDE-5I at a single andrology clinic were reviewed retrospectively. Total nucleated cell concentration and viability were measured in every product. International Index of Erectile Function-5 (IIEF-5), Erection Hardness Score (EHS) and penile colour Doppler ultrasonography were assessed at baseline and at 6 and 12 months. The primary outcome was an EHS of 3 or 4 at 6 months. Multivariate logistic regression and receiver operating characteristic (ROC) analysis were used to identify factors associated with response. *Results*: A total of 66 of 93 patients (71.0%, 95% CI 61.1–79.2) reached an EHS of 3 or 4 at 6 months; no patient had reached this threshold at baseline. Mean IIEF-5 rose from 10.1 at baseline to 16.2 at 6 months and 15.1 at 12 months (*p* = 0.001); peak systolic velocity and end-diastolic velocity both improved at 6 months (*p* = 0.001 for both). Non-responders were older (63.8 vs. 52.7 years), had longer ED duration (6.7 vs. 3.0 years), higher HbA1c (6.8 vs. 6.3) and more diabetes (66.7% vs. 42.4%). Product cell concentration (2.5 vs. 1.8) and viability (88.0% vs. 82.5%) were higher in responders (*p* = 0.001 for both). On multivariate analysis, older age (OR 1.88 per year, 95% CI 1.19–2.96), higher HbA1c (OR 13.23 per unit, 95% CI 1.65–105.87) and lower product cell viability (OR 0.38 per percentage point, 95% CI 0.20–0.73) were independently associated with non-response. On exploratory ROC analysis, cell viability discriminated responders from non-responders with an area under the curve of 0.925 (95% CI 0.872–0.977) at a threshold of 85.3%. *Conclusions*: In this uncontrolled retrospective series, SVF added to PDE-5I was associated with improved erectile function in approximately seven of ten patients. The cell viability of the administered product was independently associated with outcome, a finding that cannot be attributed to placebo response and that identifies product quality as a modifiable variable. These hypothesis-generating results require confirmation in controlled prospective studies.

## 1. Introduction

Erectile dysfunction (ED) is defined as the recurrent and consistent inability to achieve and sustain a penile erection sufficient for sexual intercourse [1]. Between 5% and 20% of men have moderate or severe ED [2], and prevalence increases markedly with age, comorbidity and obesity [3]. Although not life-threatening, ED is associated with loss of self-confidence, social isolation and deterioration of partner relationships [4]. Phosphodiesterase type 5 inhibitors (PDE-5I) are recommended as first-line therapy, but a substantial proportion of patients respond inadequately and seek additional options before considering penile prosthesis implantation [5].

Stromal vascular fraction (SVF) is a heterogeneous cellular concentrate obtained from adipose tissue by mechanical and/or enzymatic dissociation, containing endothelial progenitor cells, adipose-derived stromal cells, fibroblasts, pericytes and immune cells [6]. In a rat model of cavernous nerve injury, intracavernous SVF produced significant cavernous endothelial regeneration [7], and in the only published phase I trial in humans, Haahr and colleagues reported that 8 of 11 continent men regained the ability to have intercourse after a single intracavernous injection of adipose-derived regenerative cells [8].

Despite growing clinical use, the human evidence base remains very small and consists largely of case series and abstracts. A systematic review of cell-based therapy in male sexual dysfunction identified only one phase I trial in ED and specifically identified the absence of analysis of the SVF product itself and the short duration of follow-up as the principal weaknesses of the available literature [9]. The European Society for Sexual Medicine has likewise concluded that the current evidence is insufficient to recommend cell therapy for male sexual dysfunction outside research settings [10]. This matters because SVF is prepared at the point of care and its cellular composition varies between patients and preparations; if that variability influences outcome, it is a modifiable determinant of treatment success rather than a fixed patient characteristic.

We therefore designed this study around the product rather than the procedure. In a consecutive series of patients receiving SVF in addition to PDE-5I, total nucleated cell concentration and viability were measured in every administered product and related to clinical outcome at 6 and 12 months. The primary aim was not to demonstrate the efficacy of SVF over PDE-5I alone, which would require a controlled design, but to characterise the factors that predict response, and, in particular, to test whether the cellular quality of the administered product is one of them.

## 2. Materials and Methods

### 2.1. Study Design and Ethics

This single-centre retrospective observational study reviewed the records of consecutive patients with ED who underwent autologous SVF injection in addition to PDE-5I at a private urology and andrology clinic in Istanbul, Türkiye, between January 2022 and August 2025. All procedures were performed by two experienced andrologists at the same centre.

The study was approved by the Non-Interventional Clinical Research Ethics Committee of Haseki Training and Research Hospital, Istanbul, Türkiye (decision no. 187-2026, dated 1 July 2026). The study was conducted in accordance with the Declaration of Helsinki and is reported in accordance with the STROBE statement for observational studies. All procedures were performed in accordance with the Declaration of Helsinki. The product was autologous and minimally manipulated: it was prepared mechanically at the point of care, without enzymatic digestion, cell expansion, culture or cryopreservation, using a medical device registered with the Turkish Ministry of Health, and was returned to the same patient within the same operative session. Written informed consent for the procedure and for the research use of the resulting clinical data was obtained from every patient before treatment. As the design is retrospective, ethics approval was obtained after the treatments had been delivered.

Written informed consent was obtained from every patient before the procedure. In addition to consent for the intervention itself, the institutional consent form recorded the patient’s agreement that clinical data arising from their treatment might be used for scientific research; no patient was included whose consent form did not contain this provision.

The product was autologous, prepared at the point of care in a closed single-use system by mechanical dissociation without enzymatic digestion, and re-administered to the same patient within the same operative session. No cell expansion, culture or cryopreservation was performed at any stage. All procedures were carried out as part of routine clinical care.

### 2.2. Patients

**Figure 1:**
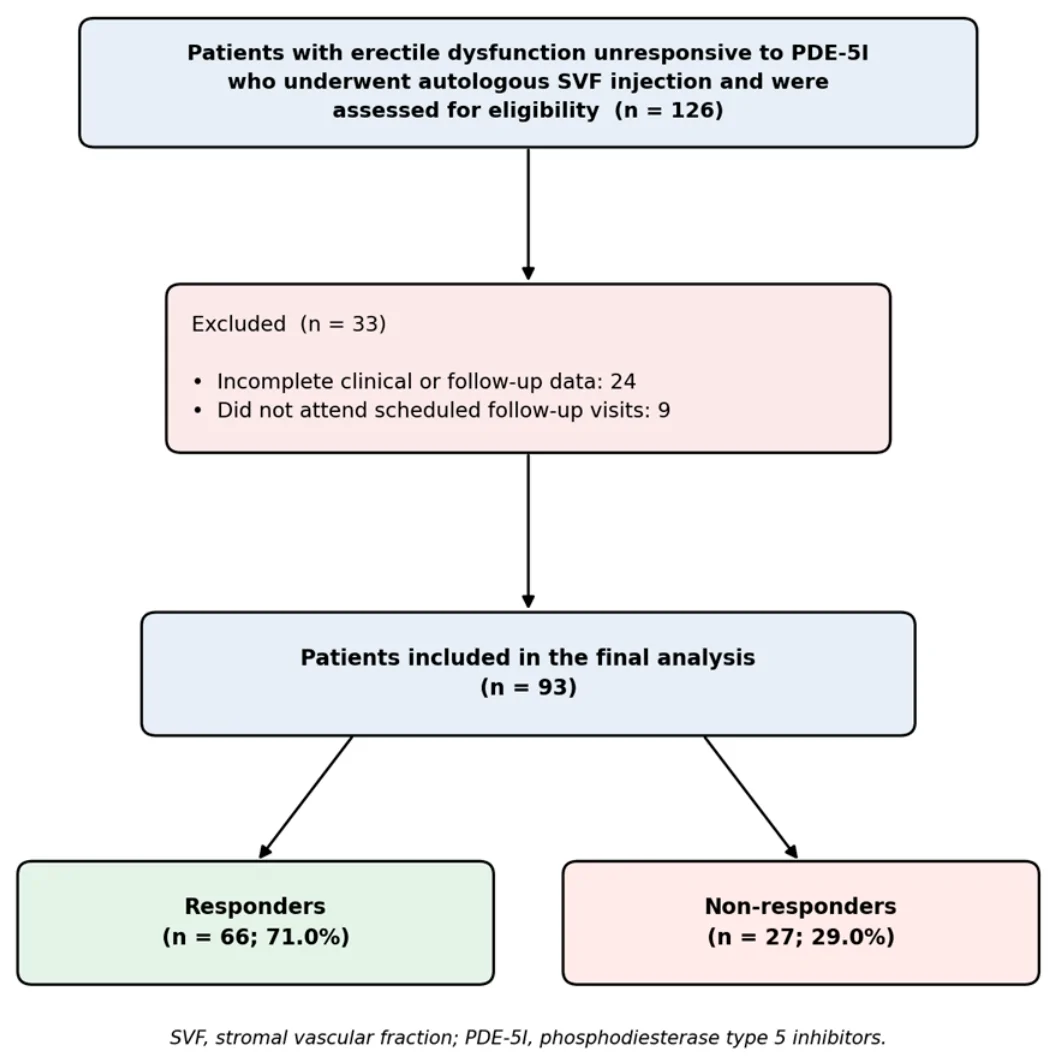
Flow diagram of patient selection. Of 126 patients assessed for eligibility, 33 were excluded according to the predefined criteria, and 93 were included in the final analysis.

A total of 126 patients treated during the study period were assessed for eligibility. Thirty-three were excluded: 24 because clinical or follow-up data were incomplete and 9 because they did not attend the scheduled follow-up visits. The remaining 93 patients constituted the study cohort (Figure 1).

Men aged 40–75 years with ED of at least one year’s duration and an IIEF-5 score below 21 were eligible. All patients had vasculogenic ED confirmed by penile colour Doppler ultrasonography, which was performed with a LOGIQ S7 Expert system (GE Healthcare, Milwaukee, WI, USA) and an ML6-15 matrix linear transducer. Following intracavernous injection of 20 µg alprostadil, the cavernous arteries were interrogated at 5, 10 and 20 min, and the highest peak systolic velocity and the lowest end-diastolic velocity recorded across these time points were used for analysis, in accordance with accepted standards for penile duplex ultrasonography. Vasculogenic ED was defined as arterial insufficiency (peak systolic velocity, PSV < 30 cm/s) or venous leakage (end-diastolic velocity, EDV > 5 cm/s), together with an inadequate response to oral PDE-5I after at least four weeks of maximum-dose therapy or intolerance due to adverse effects.

Patients with untreated severe hypogonadism (total testosterone < 300 ng/dL), uncontrolled diabetes mellitus (HbA1c > 8.5%), unstable cardiovascular disease, previous radical prostatectomy or pelvic radiotherapy, active malignancy, autoimmune disease, immunosuppressive therapy, severe coagulopathy or active skin infection at the injection site were excluded. All patients were informed in detail about the rationale, mechanism, alternatives, possible complications and follow-up schedule, and gave written consent 24 h before the procedure.

### 2.3. SVF Isolation

Autologous SVF was prepared with the GCell system (Global Biyomedikal Sağlık Hizmetleri San. ve Tic. Ltd., Şti., Istanbul, Türkiye), comprising the GCell Tissue Dissociation and Homogenization Device (reference GLB-M-1; Class IIa; GMDN 47500) and its sterile single-use tissue separation and homogenization containers (references GC-HOM-01, GC-H-01 and GC-F-01; Class IIa; GMDN 47501). All components are registered medical devices in the Turkish Medical Device Registry of the Ministry of Health (unique device identifier 8682328745003; Ministry of Health certificate no. 73795), and the containers carry CE marking under Council Directive 93/42/EEC, Annex V (EC certificate M.2020.106.13280; notified body UDEM, Ankara, Türkiye). The system operates as a closed mechanical separator without collagenase, in accordance with the principle of minimal manipulation. Approximately 15–20 mL of adipose tissue was aspirated from the periumbilical region through fine cannulas under local tumescent anaesthesia. The lipoaspirate was transferred into a sterile single-use kit without exposure to the external environment, where micro-blades and a filter system applied mechanical shear force to homogenise the tissue and release SVF cells from the extracellular matrix. The suspension was then treated using the photobiomodulation function of the device. Total preparation time was approximately 40 min.

The prepared product was administered as a single session of intracavernous injection: a total volume of 4 mL was delivered as 2 mL into each corpus cavernosum. A penile clamp was then applied for 20 min to limit systemic washout and to prolong contact between the cells and the cavernous tissue. No patient received a repeat session during the study period. Routine daily PDE-5I therapy was continued for the first three months after the procedure as part of standard post-procedural management and was then discontinued by protocol in every patient. Thereafter no patient was prescribed regular PDE-5I therapy; on-demand use during the remainder of follow-up was not systematically recorded and cannot be excluded. The 6- and 12-month assessments were therefore made after routine PDE-5I therapy had been withdrawn.

### 2.4. Cell Counting and Viability

A sample of the final product was incubated with an equal volume of 0.4% Trypan Blue. Non-viable cells with compromised membrane integrity stained blue while viable cells remained unstained. Total nucleated cell concentration (×10^6^ cells/mL) and viability (%) were determined with an automated cell counter. Measurements were performed on every product before administration, and the operator was not involved in outcome assessment.

### 2.5. Outcome Measures

The primary outcome was an Erection Hardness Score (EHS) of 3 or 4 at 6 months, a validated single measure denoting an erection sufficient for vaginal penetration. Secondary outcomes were the change in IIEF-5, the change in Doppler parameters between baseline and 6 months, and the EHS category at 12 months. Patients reaching this threshold were also required, by definition, to have been assessed on IIEF-5 and Doppler at the same visit; these measures are reported separately as secondary outcomes. The identification of factors associated with response, including the cellular characteristics of the administered product, was a prespecified secondary aim. Comparisons of baseline demographic and comorbidity variables between responders and non-responders were exploratory. Adverse events were recorded as a safety outcome.

Clinical response was assessed at baseline and at 6 and 12 months. Response was defined as all of the following: an increase of at least 4 points in IIEF-5 from baseline; an EHS of 3 or 4, sufficient for vaginal penetration; and Doppler findings indicating PSV above the arterial insufficiency threshold (>30 cm/s) and/or EDV below 5 cm/s. Patients reaching an EHS of 3 or 4 at 6 months were classified as responders and the remainder as non-responders. In this cohort, every patient who reached this threshold also showed an increase of at least 4 points in IIEF-5 and Doppler findings above the arterial insufficiency threshold and/or an end-diastolic velocity below 5 cm/s; no patient met one criterion in isolation.

### 2.6. Statistical Analysis

Analyses were performed with IBM SPSS Statistics, version 27.0 (IBM Corp., Armonk, NY, USA). Normality was assessed with histograms, probability plots and the Shapiro–Wilk test. Normally distributed continuous variables are presented as means ± standard deviation and compared with the independent samples *t*-test; categorical variables as number (percentage) and compared with the chi-square or Fisher’s exact test. Changes in IIEF-5 across the three time points were analysed with repeated-measures analysis of variance, changes in PSV and EDV between baseline and 6 months with the paired-samples *t*-test, and changes in EHS category with the chi-square test. Each candidate variable was first examined in a separate univariable logistic regression model, and the unadjusted odds ratios are reported alongside the adjusted estimates so that confounding can be assessed. A multivariable binary logistic regression model was then fitted with non-response as the dependent variable, coded 1 for patients not reaching an EHS of 3 or 4 at 6 months and 0 for those who did; odds ratios (OR) above 1 therefore denote an increased likelihood of non-response. With 27 non-responders, the model was limited to three covariates, giving nine events per variable: age, HbA1c and product cell viability, each entered as a continuous variable on its natural scale. ED duration was not entered because of collinearity with age (r = 0.63) and with cell viability (r = −0.61); baseline IIEF-5 was not entered because it showed no independent association with the outcome. Results are reported as odds ratios with 95% confidence intervals (CI). The discrimination of product cell viability and of total nucleated cell concentration for response was assessed by receiver operating characteristic (ROC) analysis, with the area under the curve (AUC) reported with its 95% CI and the optimal threshold identified by the Youden index; these analyses were exploratory, the thresholds were derived and evaluated in the same cohort, and no internal validation by resampling was performed. A two-sided *p* value < 0.05 was considered significant. Because the study is exploratory and the comparisons between responders and non-responders span a large number of baseline variables, no formal adjustment for multiple comparisons was applied; the *p* values reported for these comparisons are nominal and should be interpreted accordingly. No a priori sample size calculation was performed, and all consecutive eligible patients treated during the study period were included.

## 3. Results

### 3.1. Patient Selection and Baseline Characteristics

Ninety-three consecutive patients were included. Mean age was 55.9 ± 8.0 years, mean ED duration 4.1 ± 2.5 years and mean baseline IIEF-5 10.0 ± 2.9. Mean cell concentration and viability of the administered products were 2.3 ± 0.4 × 10^6^/mL and 86.4 ± 3.7%, respectively (Table 1).

At baseline, no patient had an EHS of 3 or above (60 patients scored 1 and 33 scored 2). At 6 months, 66 of 93 patients (71.0%, 95% CI 61.1–79.2) reached an EHS of 3 or 4 and were classified as responders, while 27 (29.0%) did not; at 12 months the corresponding figure was 65 of 93 (69.9%, 95% CI 59.9–78.3). Body mass index, total testosterone, hypertension, hyperlipidaemia and smoking were comparable between the groups. Non-responders were significantly older, had longer ED duration, higher HbA1c and a higher prevalence of diabetes mellitus. Both cell concentration and cell viability of the administered product were significantly higher in responders, and baseline PSV was higher and EDV lower in this group (Table 2).

Mean IIEF-5 increased from 10.1 at baseline to 16.2 at 6 months and 15.1 at 12 months (*p* = 0.001). At 6 months, 32 patients (34.4%) had an EHS of 3 and 34 (36.6%) an EHS of 4; the corresponding figures at 12 months were 34 (36.6%) and 31 (33.3%). PSV rose from 20.5 to 29.1 cm/s and EDV fell from 7.4 to 4.7 cm/s at 6 months (*p* = 0.001 for both) (Table 3).

The three components of the composite response definition were concordant in every patient: all 66 patients who reached an EHS of 3 or 4 at 6 months also met the IIEF-5 and Doppler criteria, and no patient met one criterion in isolation. The three domains therefore moved together in this cohort, and the single validated criterion used here captures the same patients.

On univariable analysis, older age, longer ED duration, diabetes mellitus, higher HbA1c, lower baseline IIEF-5, lower baseline PSV, higher baseline EDV, and lower product cell concentration and viability were each associated with non-response, whereas body mass index, total testosterone, hypertension, hyperlipidaemia and smoking were not (Table 4). On multivariable logistic regression with non-response as the dependent variable, older age (OR 1.88 per year, 95% CI 1.19–2.96; *p* = 0.007), higher HbA1c (OR 13.23 per unit, 95% CI 1.65–105.87; *p* = 0.015) and lower product cell viability (OR 0.38 per percentage point, 95% CI 0.20–0.73; *p* = 0.004) were each independently associated with non-response (Table 4). Each additional percentage point of cell viability was associated with a 62% reduction in the odds of non-response. The confidence interval for HbA1c is wide, and this estimate should be read as indicating direction rather than magnitude.

**Figure 2:**
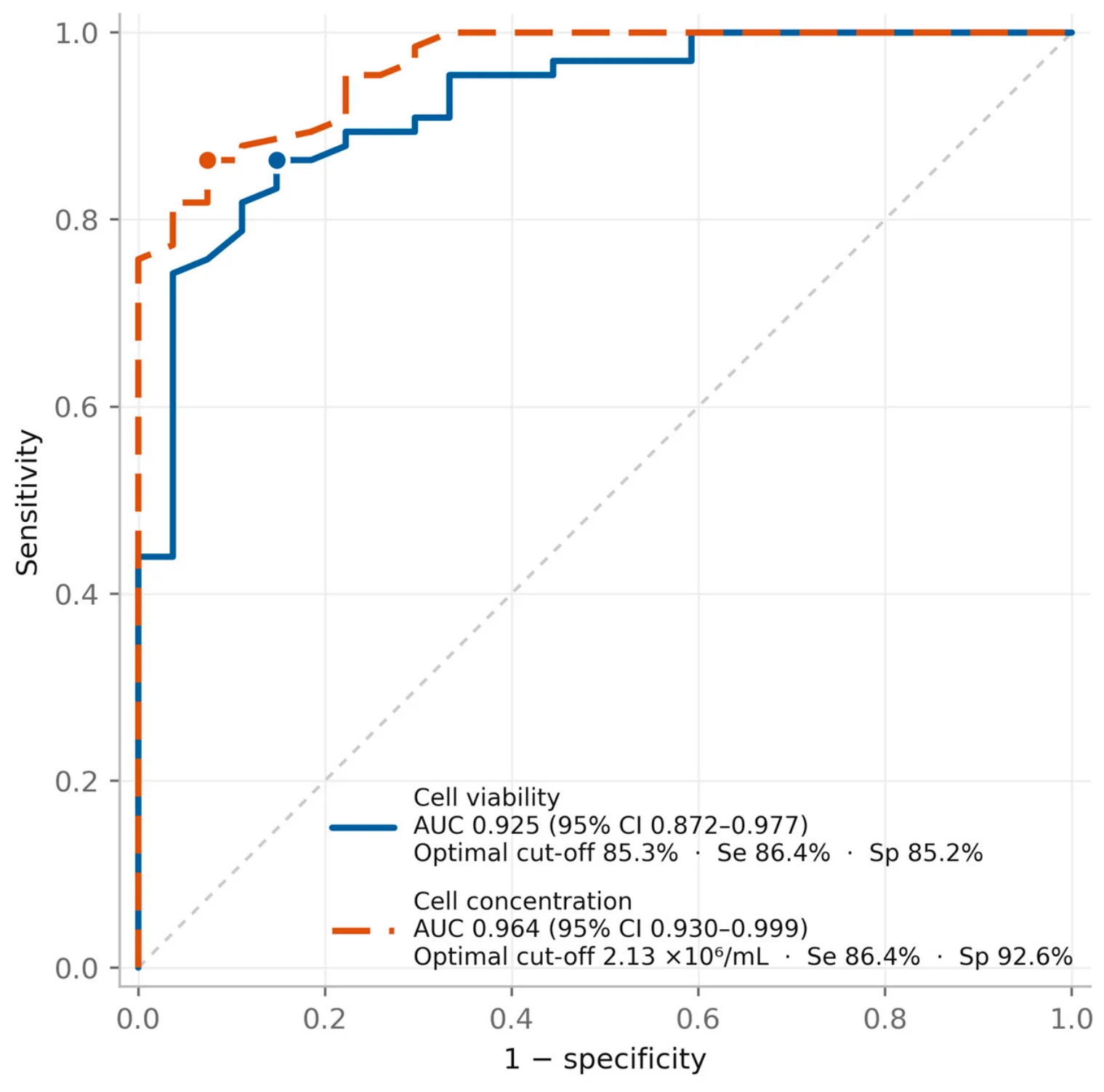
Receiver operating characteristic curves for product cell viability and total nucleated cell concentration in predicting response at 6 months (n = 93). Markers indicate the Youden-optimal point. The analysis is exploratory, and the thresholds were not internally validated.

On ROC analysis, product cell viability discriminated responders from non-responders with an AUC of 0.925 (95% CI 0.872–0.977; *p* < 0.001). The Youden-optimal threshold was 85.3%, with a sensitivity of 86.4% and a specificity of 85.2%; 13 of 93 patients were misclassified at this threshold. Total nucleated cell concentration performed at least as well (AUC 0.964, 95% CI 0.930–0.999; optimal threshold 2.13 × 10^6^ cells/mL, sensitivity 86.4%, specificity 92.6%) (Figure 2). These thresholds were derived and evaluated in the same cohort without internal validation and are therefore optimistic; they are presented as hypotheses for prospective testing and as a practical starting point for quality control of the preparation, not as validated decision rules.

### 3.2. Safety

No major complication occurred in any patient. Adverse events were confined to the donor site and to the injection procedure and were self-limiting in every case: ecchymosis at the lipoaspiration site was recorded in 21 patients (22.6%), transient pain in 19 (20.4%) and a haematoma in 1 (1.1%). All resolved without surgical intervention or hospitalisation. No infection, priapism, corporal fibrosis, penile deformity or systemic adverse reaction was observed during the 12-month follow-up (Table 5).

## 4. Discussion

In this consecutive series of 93 patients with vasculogenic ED unresponsive to PDE-5I, erectile function improved on all three measures after the addition of SVF, and approximately seven of ten patients reached an EHS of 3 or 4 at 6 months. Because the series is uncontrolled, these changes cannot be attributed to SVF. The principal finding, however, is not the response rate but its determinants: older age, higher HbA1c and, most notably, lower cell viability of the administered product were independently associated with non-response.

The absence of a control group makes it necessary to consider what might have been expected without the intervention. The placebo response in ED is substantial: a meta-analysis of 63 randomised placebo-controlled trials in 12,564 men found a significant placebo effect on erectile function (Hedges g = 0.35) [11]. Sham arms of trials of regenerative therapies improved by 0.08 to 3.8 points on the erectile function domain [12], and about one quarter of patients in placebo arms of platelet-rich plasma trials reached the minimal clinically important difference at six months [13]. The most demanding comparison concerns PDE-5I non-responders specifically: counselling and dose adjustment alone raised the mean IIEF score from 10.96 to 17.82 in 220 men who had failed sildenafil [14], and 31 of 100 apparent non-responders were converted to responders by instruction and dose optimisation alone [15]. Three features of the present cohort bear on this. Eligibility required an inadequate response after at least four weeks of maximum-dose therapy, so the dose optimisation step that accounts for most of that conversion had already been undertaken. Routine daily PDE-5I therapy was withdrawn at three months by protocol, so the 6- and 12-month assessments followed withdrawal of regular therapy, although on-demand use thereafter was not recorded and cannot be excluded. And the changes observed were not confined to a questionnaire: peak systolic velocity rose from 20.5 to 29.1 cm/s and end-diastolic velocity fell from 7.4 to 4.7 cm/s. Counselling, expectation and dose adjustment do not alter cavernous arterial haemodynamics. None of this substitutes for a randomised comparison, and we do not present it as such.

One further caveat concerns how the change in IIEF-5 should be interpreted. Published minimal clinically important differences of 2, 5 and 7 points for mild, moderate and severe ED were derived for the six-item erectile function domain, and a recent position statement holds that they apply to that instrument rather than to the five-item version and should be demonstrated between arms rather than within a single cohort [16,17]. We therefore report the change in IIEF-5 as a continuous secondary outcome and make no claim of clinical importance on that instrument.

The product-related finding deserves emphasis because it is of a different kind from the others. Age and glycaemic control are fixed patient characteristics, whereas cell viability is a property of the preparation, measured in the laboratory before administration and independent of the patient’s expectations. An association between a laboratory measurement of the administered product and the subsequent clinical outcome cannot plausibly be explained by placebo response, by regression to the mean or by observer bias, and it constitutes a dose–response-like relationship of the kind that supports causal interpretation in uncontrolled studies. To our knowledge, this is the first report in which the cellular composition of SVF has been measured in every patient and related to outcome; the absence of such analysis was identified as the main deficiency of the existing literature in a previous systematic review [9].

Penile ageing is accompanied by reduced functional capacity of corporal smooth muscle and by vascular wall damage, and the regenerative capacity of tissue and endothelium declines with age [18,19]. Gareri and colleagues found the response to PDE-5I to be significantly poorer in elderly than in younger men [20], and Albersen et al. attributed the limited benefit of PDE-5I in older men to reduced rho-kinase inhibitor, guanylate cyclase activator and maxi-K channel opener activity [21]. Our finding extends this pattern to cell-based therapy: the same biological ageing that limits pharmacological response also appears to limit regenerative response.

Viable SVF cells are thought to act largely through paracrine signalling rather than through engraftment, secreting angiogenic and anti-inflammatory mediators that promote endothelial repair and neovascularisation [22]. Injected adipose-derived stem cells leave the penis within days while functional recovery persists, and cell lysate produces recovery equivalent to that of intact cells in animal models [23,24]; human autopsy data show donor-cell persistence at very low levels that declines with time since infusion [25]. The mediators implicated in cavernous tissue include vascular endothelial growth factor, basic fibroblast growth factor, hepatocyte growth factor and insulin-like growth factor 1, together with homing through the SDF-1/CXCR4 axis and the transfer of extracellular vesicles. On this reading, viability measures how much of the secretory apparatus arrives intact, which offers a coherent explanation for the association reported here. Cell quality has been proposed as a critical determinant of the efficacy of cell-based therapy for ED [26]. Because SVF is prepared at the point of care, viability is influenced by harvesting technique, processing time, mechanical shear and temperature, all of which are under the operator’s control. If our observation is confirmed, standardisation and routine quality control of the product become as important as patient selection, and the reporting of product characteristics should become a minimum requirement in future studies of SVF. This variability is well documented: SVF is a heterogeneous population of adipose-derived stromal cells, endothelial progenitors, pericytes and leukocytes [27], and across published isolation protocols, reported viability has ranged from 39% to 98% [28]. Systematic reviews of cell therapy for ED have likewise identified the absence of standardised product characterisation as a principal obstacle to interpreting the literature [29].

One human observation is closely aligned with the present finding. In adipose-derived regenerative cell products prepared for 15 men with ED, the number of injected CD31-positive cells correlated with the erectile function outcome (r = 0.52, *p* = 0.049), whereas the total cell number and the CD34-, CD73- and CD90-positive counts did not; conditioned medium from the CD31-positive fraction produced significantly greater sprouting in human corpus cavernosum assays than that from the parent population [30]. That a property of the product, rather than the crude number of cells delivered, predicted the response supports the interpretation advanced here. Our own study measured viability and concentration only; no growth factor, cytokine or activation marker was assayed, and the mechanistic account above is therefore a hypothesis rather than a demonstrated pathway.

Men with diabetes are at higher risk of ED, and smooth muscle loss, fibrosis and microangiopathy are more frequent in this group; HbA1c reflects cumulative glycaemic exposure over approximately three months [31]. Vickers and Satyanarayana reported PDE-5I efficacy of 50–60% in diabetic compared with over 80% in non-diabetic patients [32], and Albarakati et al. found that higher HbA1c adversely affected PDE-5I efficacy [33]. The present study extends this association to SVF, suggesting that the microvascular substrate required for a regenerative response is itself compromised by poor glycaemic control.

The safety profile observed here was favourable: no major complication occurred, and the adverse events recorded were confined to donor-site ecchymosis, transient procedural pain and a single haematoma, all of which resolved without intervention. This is consistent with the small published human experience and with the minimally manipulated, autologous nature of the product. It should be noted, however, that adverse events were retrieved retrospectively from the medical records rather than collected prospectively with a structured instrument, so that minor or transient events may have been under-recorded.

Several limitations must be acknowledged. First and most importantly, the study is uncontrolled: without a comparator arm, the improvement observed cannot be attributed to SVF, and we make no claim of efficacy. Establishing efficacy relative to other second-line treatments requires a prospective randomised design and cannot be achieved by any uncontrolled series, including this one. A retrospective comparison was not feasible, since no comparable cohort receiving an alternative second-line therapy was managed at the same centre under the same follow-up schedule during the study period, and a satisfaction instrument was not part of routine documentation and cannot be reconstructed retrospectively. Second, the design is retrospective and subject to the limitations that follow from it. Third, the ROC thresholds were derived and evaluated in the same cohort without internal validation and are therefore optimistic. Fourth, the confidence interval for HbA1c in the multivariate model is wide. Fifth, adverse events were retrieved from records rather than collected prospectively. Sixth, product characterisation was limited to cell concentration and viability, with no assessment of growth factors, cytokines or cellular subpopulations. Finally, the study was conducted at a single centre by two operators, which limits generalisability.

## 5. Conclusions

In this uncontrolled retrospective series, erectile function improved in approximately seven of ten patients who had not responded to PDE-5I alone after the addition of SVF. Because there was no comparator arm, this improvement cannot be attributed to SVF, and the series does not address whether SVF is efficacious; that question requires a prospective randomised trial. Older age, higher HbA1c and lower cell viability of the administered product were independently associated with non-response. The viability finding is the most actionable: unlike patient age and glycaemic control, product quality is modifiable, and its association with outcome argues for standardised preparation and routine reporting of SVF product characteristics. These findings are hypothesis-generating and require confirmation in prospective controlled studies before SVF can be recommended for routine clinical use in this setting.

## Figures and Tables

**Table 1:** Baseline demographic and clinical characteristics of all patients (n = 93).

Variable	Value
Age (years) *	55.9 ± 8.0
Body mass index (kg/m^2^) *	27.6 ± 4.2
ED duration (years) *	4.1 ± 2.5
Diabetes mellitus, n (%)	46 (49.5)
Hypertension, n (%)	50 (53.8)
Hyperlipidaemia, n (%)	45 (48.4)
Smoking, n (%)	46 (49.5)
HbA1c (%) *	6.4 ± 1.0
Total testosterone (ng/dL) *	459.1 ± 105.4
Cell concentration (×10^6^/mL) *	2.3 ± 0.4
Cell viability (%) *	86.4 ± 3.7
IIEF-5 at baseline *	10.0 ± 2.9
EHS 1 at baseline, n (%)	60 (64.5)
EHS 2 at baseline, n (%)	33 (35.5)
PSV at baseline (cm/s) *	20.5 ± 3.1
EDV at baseline (cm/s) *	7.4 ± 1.1

* Mean ± standard deviation. ED, erectile dysfunction; HbA1c, glycated haemoglobin; IIEF, International Index of Erectile Function; EHS, Erection Hardness Score; PSV, peak systolic velocity; EDV, end-diastolic velocity.

**Table 2:** Comparison of baseline characteristics between responders and non-responders.

Variable	Responders (n = 66)	Non-Responders (n = 27)	*p*
Age (years) *	52.7 ± 6.1	63.8 ± 6.4	0.001
Body mass index (kg/m^2^) *	27.2 ± 3.9	28.3 ± 4.6	0.255
ED duration (years) *	3.0 ± 1.6	6.7 ± 2.2	0.001
Diabetes mellitus, n (%)	28 (42.4)	18 (66.7)	0.034
Hypertension, n (%)	33 (50.0)	17 (63.0)	0.255
Hyperlipidaemia, n (%)	29 (43.9)	16 (59.3)	0.180
Smoking, n (%)	30 (45.5)	16 (59.3)	0.227
HbA1c (%) *	6.3 ± 0.9	6.8 ± 1.2	0.016
Total testosterone (ng/dL) *	468.2 ± 101.4	437.0 ± 113.4	0.197
Cell concentration (×10^6^/mL) *	2.5 ± 0.3	1.8 ± 0.3	0.001
Cell viability (%) *	88.0 ± 2.7	82.5 ± 2.8	0.001
IIEF-5 at baseline *	11.1 ± 2.5	7.3 ± 1.9	0.001
EHS 1 at baseline, n (%)	38 (57.6)	22 (81.5)	0.029
EHS 2 at baseline, n (%)	28 (42.4)	5 (18.5)	
PSV at baseline (cm/s) *	21.6 ± 2.7	17.7 ± 2.3	0.001
EDV at baseline (cm/s) *	7.1 ± 0.9	8.3 ± 1.0	0.001

* Mean ± standard deviation. Abbreviations as in Table 1.

**Table 3:** Functional outcomes after SVF administration.

Variable	Baseline	6 Months	12 Months	*p*
IIEF-5 *	10.1 ± 2.9 ^a^	16.2 ± 5.9 ^b^	15.1 ± 5.9 ^b^	0.001
EHS 1–2, n (%)	93 (100)	27 (29.0)	28 (30.1)	0.001
EHS 3, n (%)	0	32 (34.4)	34 (36.6)	
EHS 4, n (%)	0	34 (36.6)	31 (33.3)	
PSV (cm/s) *	20.5 ± 3.1	29.1 ± 6.6	—	0.001
EDV (cm/s) *	7.4 ± 1.1	4.7 ± 2.3	—	0.001

* Mean ± standard deviation. Superscript letters denote the source of the difference: identical letters indicate no significant difference between time points; different letters indicate a significant difference. Doppler measurements were repeated at 6 months only. Abbreviations as in Table 1.

**Table 4:** Univariable and multivariable logistic regression analysis of factors associated with non-response at 6 months (n = 93).

Variable	Unadjusted OR (95% CI)	*p*	Adjusted OR (95% CI)	*p*
Age (per year)	1.32 (1.17–1.49)	<0.001	1.88 (1.19–2.96)	0.007
Body mass index (per kg/m^2^)	1.07 (0.95–1.19)	0.253	—	—
ED duration (per year)	3.03 (1.88–4.87)	<0.001	—	—
Diabetes mellitus (present)	2.71 (1.06–6.93)	0.037	—	—
Hypertension (present)	1.70 (0.68–4.26)	0.257	—	—
Hyperlipidaemia (present)	1.86 (0.75–4.60)	0.182	—	—
Smoking (current)	1.75 (0.70–4.33)	0.229	—	—
HbA1c (per %)	1.70 (1.09–2.66)	0.019	13.23 (1.65–105.87)	0.015
Total testosterone (per 10 ng/dL)	0.97 (0.93–1.02)	0.197	—	—
Baseline IIEF-5 (per point)	0.47 (0.34–0.64)	<0.001	—	—
Baseline PSV (per cm/s)	0.57 (0.45–0.73)	<0.001	—	—
Baseline EDV (per cm/s)	3.82 (2.03–7.19)	<0.001	—	—
Product cell concentration (per 0.1 × 10^6^/mL)	0.30 (0.16–0.56)	<0.001	—	—
Product cell viability (per %)	0.49 (0.37–0.66)	<0.001	0.38 (0.20–0.73)	0.004

The dependent variable is non-response, coded 1 for patients not reaching an Erection Hardness Score of 3 or 4 at 6 months and 0 for responders; odds ratios above 1 therefore denote an increased likelihood of non-response. Unadjusted odds ratios were obtained from separate univariable logistic regression models, one per variable. The multivariable model was limited to three covariates because 27 events were available, giving nine events per variable; variables not entered in that model are shown as a dash. Continuous variables were entered on their natural scale, with the unit of increase given in parentheses. OR, odds ratio; CI, confidence interval; ED, erectile dysfunction; PSV, peak systolic velocity; EDV, end-diastolic velocity.

**Table 5:** Adverse events recorded during follow-up (n = 93).

Adverse Event	n (%)	Outcome
Ecchymosis at donor site	21 (22.6)	Resolved spontaneously
Transient procedural pain	19 (20.4)	Resolved with simple analgesia
Haematoma	1 (1.1)	Resolved without intervention
Infection	0 (0)	—
Priapism	0 (0)	—
Corporal fibrosis or penile deformity	0 (0)	—
Systemic adverse reaction	0 (0)	—
Major complication requiring surgery or admission	0 (0)	—

Adverse events were retrieved from the medical records and were not collected using a predefined structured instrument.

## Data Availability

The data presented in this study are available from the corresponding author upon reasonable request.
